# Genetic transformation of *Knufia petricola* A95 - a model organism for biofilm-material interactions

**DOI:** 10.1186/s13568-014-0080-5

**Published:** 2014-11-04

**Authors:** Steffi Noack-Schönmann, Tanja Bus, Ronald Banasiak, Nicole Knabe, William J Broughton, H Den Dulk-Ras, Paul JJ Hooykaas, Anna A Gorbushina

**Affiliations:** 1 Department 4 (Materials & Environment), Federal Institute for Materials Research and Testing (Bundesanstalt für Material-forschung und -prüfung, BAM), Unter den Eichen 87, Berlin, 12205, Germany; 2 Department of Biotechnology, University of Applied Sciences Jena, Carl-Zeiß-Promenade 2, Jena, 07745, Germany; 3 Institute of Biology Leiden, Universiteit Leiden, Sylviusweg 72, Leiden, 2333 BE, The Netherlands; 4 Department of Biology, Chemistry and Pharmacy & Department of Earth Sciences, Freie Universität Berlin, Malteserstrasse 74-100, Berlin, 12249, Germany

**Keywords:** DNA transfer, Fungal cell-walls, Protoplasts, Hygromycin resistance, Black yeast, Sub-aerial biofilms, Stress-protective morphology, Ancestor of opportunistic pathogens & lichens

## Abstract

We established a protoplast-based system to transfer DNA to *Knufia petricola* strain A95, a melanised rock-inhabiting microcolonial fungus that is also a component of a model sub-aerial biofilm (SAB) system. To test whether the desiccation resistant, highly melanised cell walls would hinder protoplast formation, we treated a melanin-minus mutant of A95 as well as the type-strain with a variety of cell-degrading enzymes. Of the different enzymes tested, lysing enzymes from *Trichoderma harzianum* were most effective in producing protoplasts. This mixture was equally effective on the melanin-minus mutant and the type-strain. Protoplasts produced using lysing enzymes were mixed with polyethyleneglycol (PEG) and plasmid pCB1004 which contains the hygromycin B (HmB) phosphotransferase (*hph*) gene under the control of the *Aspergillus nidulans trp*C. Integration and expression of *hph* into the A95 genome conferred hygromycin resistance upon the transformants. Two weeks after plating out on selective agar containing HmB, the protoplasts developed cell-walls and formed colonies. Transformation frequencies were in the range 36 to 87 transformants per 10 μg of vector DNA and 10^6^ protoplasts. Stability of transformation was confirmed by sub-culturing the putative transformants on selective agar containing HmB as well as by PCR-detection of the *hph* gene in the colonies. The *hph* gene was stably integrated as shown by five subsequent passages with and without selection pressure.

## Introduction

Melanised microcolonial fungi (MCF) are a taxonomically diverse group of Ascomycetes that colonise sub-aerial rock surfaces and other varied materials in all regions of the world, from the Antarctic to hot deserts (Staley et al. [1982]; Gorbushina et al. [1993]; Urzi et al. [1995]; Wollenzien et al. [1995]; Sterflinger and Prillinger [2001]; Ruibal [2004]; Selbmann et al. [2005]; Gorbushina [2007]; Ruibal et al. [2008]). MCF are characterised by simple stress-protective morphologies including a peculiar compact colonial structure, protective cell walls and multiple secondary metabolic products that support stress tolerance – carotenoids, melanins, mycosporines and compatible solutes (e.g. Gorbushina et al. [2008]). For all these reasons MCF are unequalled amongst eukaryotes in their ability to withstand extreme heat, desiccation and UV radiation. MCF interact with accompanying phototrophic algae, cyanobacteria and heterotrophic bacteria to form a stable sub-aerial (SABs) microbial community (Gorbushina et al. [2005]; Gorbushina [2007]). At the extreme, MCF survival can depend on the small amounts of nutrients available in atmospheric aerosols, gases and air-borne particles – as well as on other SAB inhabitants (Gorbushina [2007]). As a consequence of MCF stress tolerance, SABs are pioneer settlers on diverse surfaces exposed to air including artificial materials. Eventually SABs cause discoloration and deterioration of facades and roofs of buildings and are thus an important aspect of material science.

*Knufia petricola* A95 is a well suited model fungus to study organism-organism and organism-material interactions in SABs (Gorbushina and Broughton, [2009]; Nai et al. [2013]). As the rock-inhabiting *K. petricola* A95 belongs to an ancestral lineage of the Chaetothyriales (Ascomycetes) and is a predecessor of opportunistic pathogens and lichens (Gueidan et al. [2008]), it is also of potential importance for understanding the evolution of Ascomycete life styles. Nevertheless, to be a tractable model organism, a reliable and efficient transformation system is essential (Perez-Nadales et al. [2014]).

SABs are principal determinants of weathering of rocks (Gorbushina and Krumbein, [2000]) and other materials exposed to air (e.g. Noack-Schönmann et al. [2014]). SABs are important in the material sciences not only because they are omni-present on all exposed materials, but also because they degrade the surfaces to which they adhere. Weathering results and transparent surfaces become opaque restricting the passage of radiation (Hallbauer and Jahns, [1977]; Jones and Wilson, [1985]; Chen et al. [2000]; Avakian et al. [1981]; Jongmans et al. [1997]; Gadd et al. [1999]; Burford et al. [2003]; Gorbushina et al. [1993]; Noack-Schönmann et al. [2014]; Shirakawa, Zilles, Mocelin, Gaylarde, Gorbushina, Heidrich, Giudice, Del Negro, John. Microbial colonisation affects the efficiency of photovoltaic panels in a tropical environment, submitted to Science of the Total Environment). Diagnostic and prognostic tools for biofilm development on different materials and under various environmental conditions require easily accessible and genetically modifiable SABs. The establishment of a DNA-transfer- and integration-system into *K. petricola* A95 will set the stage for a variety of investigations into the role of MCFs in SAB formation and biofilm-induced material deterioration.

An efficient transformation system is particularly important in dual-component systems like sub-aerial biofilms (SAB) where rock-inhabiting fungi form close inter- and intra-cellular contacts with phototrophic cells (Gorbushina et al. [2005]). Gorbushina and Broughton ([2009]) proposed a model biofilm comprising the cyanobacterium *Nostoc punctiforme* and the MCF *Knufia petricola* (A95) as a new test system for the simulation of environment-biofilm interactions and their impact on materials. As an experimental model it is however essential that both partners be accessible to genetic manipulation. A transformation system exists for *N. punctiforme* (e.g. Risser and Meeks, [2013]) and here we report a method for stably transforming *K. petricola*.

## Materials and methods

### Strains and media

**Figure 1:**
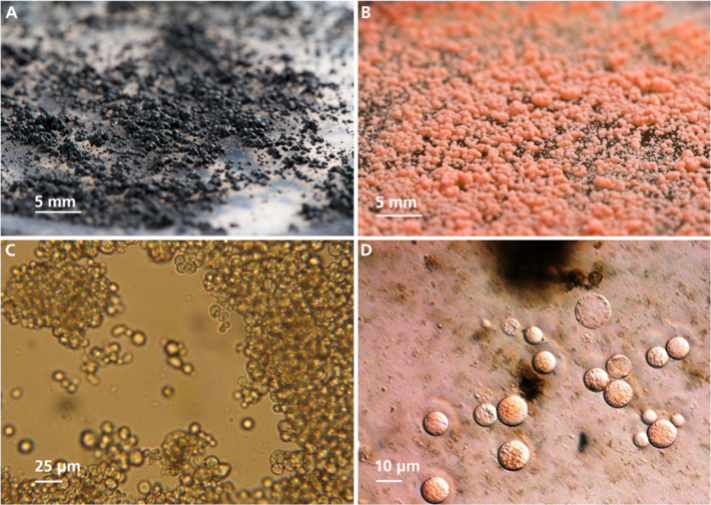
***Knufia petricola*A95 morphology. A**- Being a typical MCF, *K. petricola* A95 retains protective pigmentation and restricted colony growth even under favourable growth conditions on a Petri dish; **B** - As a result of a spontaneous mutation, the A95 pink mutant (A95p) is deficient in melanin synthesis. The orange/red colour is caused by carotenoids that are normally masked by melanins. **C**- Micrograph of A95 cells with melanin. The ball-shaped, compact A95 cells are surrounded by a thick cell-wall and are embedded in extracellular polymeric substances that are arranged in compact clusters of cells. **D**- A95 protoplasts prepared from similar cells to those shown in Figure 1C. A95 protoplasts were most effectively isolated by digestion of the cell-wall using lysing enzymes from *Trichoderma harzianum* and prevented from bursting in a 1M KCl buffer.

*K. petricola* A95 (= CBS 123872) was isolated from the surface of a marble rock near the Philopappos monument on Musaios Hill, Athens (Greece) (Gorbushina et al. [2008]). It is maintained in the culture collection of the Centraalbureau for Schimmelcultures (CBS 123872) in Utrecht and the Federal Institute for Materials Research and Testing in Berlin (A95). A spontaneous pink mutant of A95 deficient in melanin synthesis was isolated (A95p). The characteristic orange/pink colour of the mutant (Figure 1B) is caused by carotenoids (Gorbushina et al. [2008]) that are normally masked by the melanised cell-wall. The type-strain (A95) and A95p were cultivated in liquid malt-extract medium (MEB)[2% (w/v) malt extract, 0.1% (w/v) peptone, 2% (w/v) glucose] at 25°C and 100 rpm. The cultures were refreshed each week as described in Nai et al. ([2013]). A one week old culture was homogenised along with 10 steel balls (Ø 5 mm) in a ball mill (Retsch^TM^, Retsch GmbH, 42781 Haan, Germany) for 30 s^−1^ and 10 min then diluted 1:100 with fresh malt extract medium.

### Formation of protoplasts

300 ml Erlenmeyer flasks containing 100 ml of MEB were inoculated with 1 ml of one-week-old freshly dispersed A95 and A95p cultures grown in MEB (experiments were duplicated for both strains). The cultures were grown for three days at 25°C and 100 rpm, spun down at 3,000 × g for 10 min and the resulting biomass washed three times with protoplast buffer (0.01 M *Tris–HCl*, 0.01 M MgSO_4_, 1M KCl, pH 7.0 (Walz and Kück, [1995])). Different enzyme combinations were added to 2 × 10^7^ cfu A95 in 20 ml protoplast buffer. The enzymes used were: (i) a mixture of Vinoflow (Novozymes, Bagsvaerd, Denmark), Yatalase (Takara, Shiga Japan) and ß-glucanase (*Aspergillus niger*)(Sigma-Aldrich, Steinheim, Germany)(each 10 mg.ml^−1^) (ii) Lysing enzymes from *Trichoderma harzianum* (Sigma-Aldrich)(10mg.ml^−1^) and (iii) a mixture of Driselase (Sigma-Aldrich), Lyticase (Sigma-Aldrich) and ß-glucanase (*A.niger*) (Sigma-Aldrich) also at 10 mg.ml^−1^. The A95-enzyme mixes were incubated at 27°C and 100 rpm for 24 h. The efficiency of protoplast formation (ratio of protoplasts to the number of A95 cells still surrounded by cell walls) was determined in 10 μl aliquots by counting in a Neubauer chamber. For transformation, the protoplasts were centrifuged for 5 min at 3,000 × g, washed several times with transformation buffer [1M sorbitol, 80 mM CaCl_2_, pH 7.0 (Walz and Kück, [1993])] and re-suspended in the same buffer at a final concentration of 5 × 10^7^.ml^−1^.

### Recombinant plasmids

**Figure 2:**
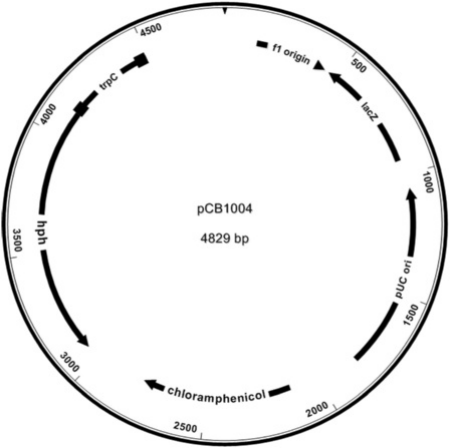
**Plasmid map of pCB1004.** Plasmid pCB1004 was constructed by Carroll et al. ([1994]). The vector has a chloramphenicol resistance- and a functional l*acZ*-gene for blue-white screening. *hph* = Hygromycin B resistance; *trpC* = *A. nidulands* promoter.

The hygromycin selection vector for PEG mediated transformation pCB1004 (Carroll et al. [1994]) was used. pCB1004 contains the hygromycin B phosphotransferase gene (*hph*) under control of the *A. nidulans trpC* promoter (Mullaney et al. [1985]) but without the *trpC* terminator. pCB1004 also contains a chloramphenicol resistance cassette, a functional *lacZ* gene and a multiple cloning site (Figure 2).

### PEG mediated transformation

50 μl (≈ 10^6^) protoplasts, re-suspended in transformation buffer, were incubated with 10–20 μg of plasmid DNA for 20 min at 4°C. 100 μl of 24% (w/v) PEG 6000 was added, gently mixed and incubated for 30 min at room temperature. Five mL MEBS [malt extract broth with 10.8% (w/v) sucrose] containing 150 μl transformation mix was distributed over five MEAS-plates [malt-extract agar with 10.8% (w/v) sucrose]. After incubation at 25°C for 24 h, the plates were overlain with 5 ml of selective agar [0.8 M NaCl, 0.4% w/v) agar, final concentration of 0.25 mg of HygB.ml^−1^, pH 7.0] and incubated for 3 to 4 weeks at 25°C.

### Isolation of fungal DNA

A95 cells were frozen in liquid nitrogen and ground with a mortar and pestle. 100 mg of the ground cells was mixed with 500 μl Genomic Lysis Buffer (OmniPrep^TM^ for Fungus Kit, G-Biosciences, St. Louis, MO 63132–1429, U.S.A.) and total DNA isolated using the same kit according to the manufacturer’s instructions. Five randomly selected A95 transformants were used for total DNA isolation and amplification of the *hph* gene.

### Amplification of the hygromycin B phosphotransferase gene (*hph*)

On the assumption that the hygromycin B phosphotransferase gene (*hph*) was integrated randomly into the genome of A95, total genomic DNA of the transformants was amplified with the primer pair hphRforw (5’-ATGCCTGAACTCACCGCGAC-3’) and hphRrev (5’-CTATTCCTTTGCCCTCGGACGAG-3’). PCR reactions were carried out with *ReproFast*-DNA Polymerase (Genaxxon Bioscience, 89007 Ulm, Germany) under the following conditions: initial denaturation at 96°C for 30s, followed by 30 cycles of denaturation at 96°C for 30s, annealing at 54°C for 30s, extension at 72°C for 2 min, terminated by 10 min extension at 72°C. The PCR products were sequenced.

### Mitotic stability of the A95 transformants

Five randomly selected colonies were transferred to 5 ml MEA, mixed with 10 steel balls (Ø 5 mm), vigorously shaken in a Retsch^TM^ mill for 10 min at 30 Hz and subcultured on MEA with and without hygromycin B. The putative transformants were subcultured five times on selective or non-selective agar. After five rounds of non-selective subculture, the transformants were transferred to MEA plates containing 50 μg.ml^−1^ hygromycin B. This experiment was repeated twice.

## Results

Different ways of transforming A95 were tested (S. Noack-Schönmann et al. unpublished results). These included the commonly used binary Ti-vector system of *A. tumefaciens* (De Groot et al. [1998], Michielse [2005a]; [2005b]; [2008]) which failed to yield transformants in our hands. As A95 is strongly melanised, problems in penetrating the melanised and as a consequence thick cell wall were expected. Chemical weakening of the cell wall with DMSO before addition of *A. tumefaciens* was also unsuccessful. Bombardment of A95 with micro-projectiles (Lorito et al. [1993]; Te’o et al. [2002]) coated with pTAS5 and pCB1004 plasmid DNA did not yield transformants. Finally different enzyme preparations containing various chitinases and β-glucanases were tested to establish an efficient protocol for the isolation of protoplasts. Armed with an effective protocol for the production of protoplasts, we used the vector pCB1004 carrying the hygromycin resistance gene and polyethylene glycol to transfer hygromycin resistance to A95.

### Protoplast isolation

All enzymes and enzyme mixtures tested yielded protoplasts (Figure 1D and Table 1). At best, the protoplast yields were ten percent of the intact cells subjected to the enzyme treatment (Table 1). Clearly, the mixture Driselase, ß-Glucanase and Lyticase was only about half as efficient as the other two treatments. On the other hand, there were no significant differences in protoplast yields obtained with the lysing enzymes of *T. harzianum* and the mixture ß-Glucanase, Vinoflow and Yatalase. As preparation of the *T. harzianum* mix is simpler and more cost effective, we used this procedure in all further experiments. Black A95 cells (A95) and cells of a pink melanin-deficient A95 mutant (A95p) (Figure 1) were subjected to treatment with lysing enzymes of *T. harzianum* as described above. Although higher than in preliminary experiments, protoplast yields were the same for both cell types (Table 2).

**Figure 3:**
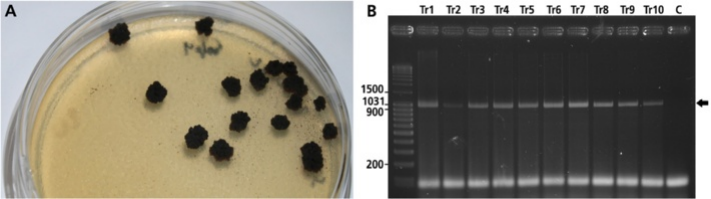
**A95 transformants. A**- three weeks after PEG mediated transformation of A95 protoplasts with pCB1004, single A95 colonies were observed on malt-extract agar plates containing hygromycin. **B**- PCR products of the *hph*-gene (1,019 bp) seen on agarose gels. Polymerase chain reaction detection of the *hph*-gene from DNA of five independent putative transformants confirmed integration of *hph* into the genome of A95 transformants. Arrow indicates location of *hph* DNA bands.

**Table 1:** Efficiency of protoplast isolation from A95 cells using different cell-wall lysing enzymes

**Enzymes used for protoplast isolation**	**Starting number of A95 cells (cfu.mL**^**−1**^**)**	**Number of protoplasts isolated.mL**^**−1**^
Lysing enzymes from *Trichoderma harzianum*	2 × 10^7^	2.0 × 10^6^
β-Glucanase, Vinoflow, Yatalase	2 × 10^7^	1.9 × 10^6^
Driselase, β-Glucanase, Lyticase	2 × 10^7^	1.1 × 10^6^

**Table 2:** Efficiency of protoplast isolation from A95 and a pink A95 derivative

	**Starting number of cells (cfu.mL**^**−1**^**)**	**Number of protoplasts isolated.mL**^**−1**^
A95	5 × 10^6^	1.1 × 10^6^
Pink derivative of A95	5 × 10^6^	1.1 × 10^6^

### Transformation of A95 protoplasts

Colonies were visible on hygromycin B containing MEAS agar two to three weeks after transformation of A95 protoplasts with pCB1004 (Figure 3A). Five randomly selected, transformed colonies were able to grow when plated out again on hygromycinB-containing agar. Total DNA from some of these re-picked colonies was amplified by PCR using the primer pair hphRforw/hphRrev resulting in an amplicon of ≈ 1,000 bp, which is consistent with the size (1,020 bp) of the *hph*-gene (Figure 3B). The sequences of all PCR products matched completely those of *hph*. About 55 transformants were obtained from 10^6^ protoplasts in a total volume of 150 μl containing 10 to 20 μg plasmid DNA.

### Mitotic stability

All randomly selected A95 transformants maintained hygromycin resistance after multiple passages on MEA with or without hygromycin. After subculturing five times without selection pressure (MEA without hygromycin), five formerly hygromycin-resistant colonies were still able to grow when plated out again on hygromycin containing MEA.

## Discussion

An assembly of methods that are used for transformation of filamentous fungi were tested in preliminary experiments, but only PEG-mediated transformation of protoplasts showed promise. We performed PEG-mediated transformation of A95 protoplasts with pTAS5 (de Groot et al. [1998]) and pCB1004 (Carroll et al. [1994]), but only obtained transformants using pCB1004. Perhaps the TAS5 vector (11.8 kb) was too large to escape restriction on entering the A95 protoplasts or the *gpd*A promoter was less efficient in A95 than the *trp*C promoter.

Incubation of protoplasts with pCB1004 and PEG allowed integration of the hygromycin B resistance cassette (under control of the *trp*C promoter) into the genome of A95. All hygromycin B resistant colonies selected maintained resistance to hygromycin during five rounds of subculturing on selective or non-selective agar. Most probably transformation efficiencies can be improved by for example optimising the ratio between applied plasmid-DNA and the number of cells. Various preparations containing a complex mixture of hydrolytic enzymes were tested (Table 1) for preparation of transformable protoplasts. Lysing enzymes of *Trichoderma harzianum* were most effective in isolating protoplasts from A95 cells (Table 1). Further improvement of the protoplast isolation protocol by testing different enzyme concentrations and various incubation temperatures could also be tried. Interestingly, melanin from the pigmented walls did not interfere with protoplast isolation since the efficiency of protoplast formation was the same using type A95 cells and a melanin-deficient mutant (Table 2).

Stable integration of the hygromycin-resistance gene into the A95 genome showed that illegimate recombination is possible in *Knufia petricola* A95. Whether this means that homologous recombination also occurs at a frequency that would permit gene-replacement remains to be determined. Nevertheless, the methods described here permit exploration of A95 using reverse genetic tools. Armed with these tools, it should be possible to discover the mechanisms which A95 and other rock-inhabiting melanised fungi use to colonise inhospitable sub-aerial surfaces so successfully.

## Competing interests

The authors declare that they have no competing interests.

## Authors’ contributions

SNS performed many types of experiments to produce protoplasts and transform them, actively participated in the design of the study and drafted a preliminary manuscript containing Figures 1 and 3. TB prepared many batches of protoplasts. NK conducted the transformation experiments and prepared Figures 2 and 3. RB assisted in all experiments conducted at the BAM and attempted transformation using the “GeneGun”. HDDR, PJJH and WJB initiated protoplast isolation and *Agrobacterium*-mediated transformation experiments in the Leiden laboratories. They also participated in the design of later experiments. WJB shaped the presentation of the material written. AAG conceived the study, participated in its design and coordination, helped to interpret the data and write the manuscript. All authors read and approved the final manuscript.
